# Fucose as a nutrient ligand for Dikarya and a building block of early diverging lineages

**DOI:** 10.1186/s43008-023-00123-8

**Published:** 2023-09-05

**Authors:** Małgorzata Orłowska, Drishtee Barua, Sebastian Piłsyk, Anna Muszewska

**Affiliations:** 1 Institute of Biochemistry and Biophysics, Polish Academy of Sciences, Pawinskiego 5A, 02-106 Warsaw, Poland; 2 Institute of Evolutionary Biology, Faculty of Biology, Biological and Chemical Research Centre, University of Warsaw, Zwirki i Wigury 101, 02-089 Warsaw, Poland

**Keywords:** Fucosyltransferases, Fucose degradation, Fungal proteins, Fungal enzymes, Lectins, Fucose biosynthesis, Protein family expansion, Mucoromycotina, Fucose metabolism

## Abstract

**Abstract:**

Fucose is a deoxyhexose sugar present and studied in mammals. The process of fucosylation has been the primary focus in studies relating to fucose in animals due to the presence of fucose in Lewis antigens. Very few studies have reported its presence in Fungi, mostly in *Mucoromycotina*. The constitution of 25% and 12% of this sugar in the carbohydrates of cell wall in the respective *Umbelopsis* and *Mucorales* strains boosts the need to bridge the gap of knowledge on fucose metabolism across the fungal tree of life. In the absence of a network map involving fucose proteins, we carried out an *in-silico* approach to construct the fucose metabolic map in *Fungi*. We analyzed the taxonomic distribution of 85 protein families in *Fungi* including diverse early diverging fungal lineages. The expression of fucose-related protein-coding genes proteins was validated with the help of transcriptomic data originating from representatives of early diverging fungi. We found proteins involved in several metabolic activities apart from fucosylation such as synthesis, transport and binding. Most of the identified protein families are shared with *Metazoa* suggesting an ancestral origin in *Opisthokonta*. However, the overall complexity of fucose metabolism is greater in Metazoa than in *Fungi*. Massive gene loss has shaped the evolutionary history of these metabolic pathways, leading to a repeated reduction of these pathways in most yeast-forming lineages. Our results point to a distinctive mode of utilization of fucose among fungi belonging to *Dikarya* and the early diverging lineages. We speculate that, while *Dikarya* used fucose as a source of nutrients for metabolism, the early diverging group of fungi depended on fucose as a building block and signaling compound.

**Graphical abstract:**

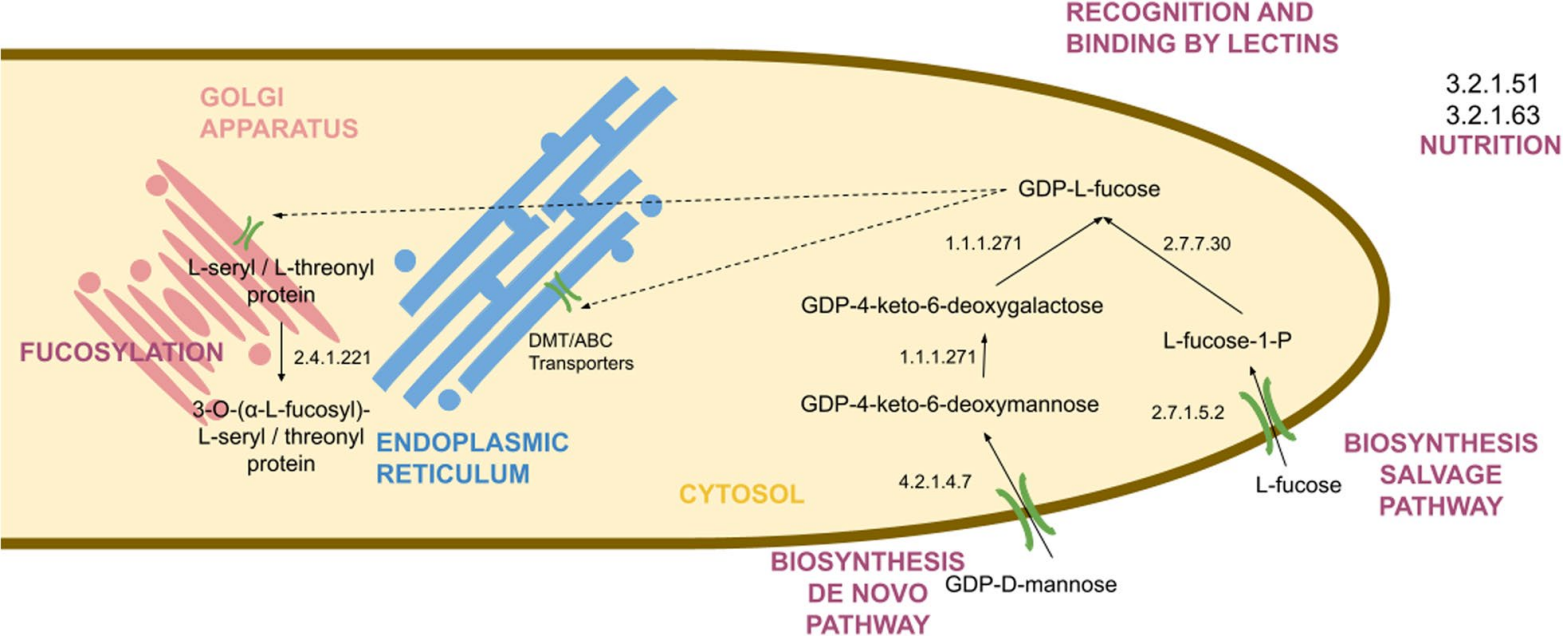

**Supplementary Information:**

The online version contains supplementary material available at 10.1186/s43008-023-00123-8.

## INTRODUCTION

Fucose (6-deoxy hexose in the L-configuration) is a relatively less abundant carbohydrate compared to other simple sugars. It can bind to glycans, glycoproteins and glycolipids (Ma et al. 2006). All domains of life contain fucosylated molecules either on the surface of the cell in the form of cell wall polysaccharides in fungi and plants, lipopolysaccharides in bacteria, ABO group antigens in mammals or within the cell. The fucosylation process and its role, as well as the general fucose metabolism, are best described in mammals (Kizuka et al. 2017). However, studies on fucose biology have also been carried out for *Caenorhabditis* (Yan et al. 2015, 2018) and *Drosophila* (Ishio et al. 2015). To date, three main fucosylation mechanisms can be distinguished. Terminal fucosylation and core fucosylation of N-linked glycans are common and well-described types of fucose modification, in contrast to O-fucosylation that has been well-characterized only in mammals and in *Helicobacter pylori (*Schneider et al. 2017*)*. L-Fucose can be bound to serine and threonine residues via* O*-fucosylation and act as a signaling molecule. Fucose can also be linked to *N*-glycans via α-linkage to galactose or *N*-acetylglucosamine (GlcNAc) residues. Such structures are involved in various recognition processes, including fertilization and development, immunological response, cancer antigens hiding or during apoptosis (Staudacher et al. 1999).

The greatest emphasis has been put on studying the role of fucose in mammals. Some fucosyltransferases are known to be essential for the proper gastrulation of mice (Du et al. 2010; Schneider et al. 2017). Fucose is also an important compound in the human gastrointestinal tract as part of mucus glycans and blood group ABO and Lewis antigens (Garber et al. 2021). Fucosylation plays a role in the regulation of immunity and cancer development, synapse formation along with fertilization and host-microbe interactions (Schneider et al. 2017). The best-known interaction mediated by this kind of modification is the colonization of human gastric mucosa by the bacterium *H. pylori.* This microbe can mimic human Lewis antigen to evade the host immune response (Moran 2008).

So far a few reports on fungal proteins acting on fucose (and perhaps arabinose (Ogawa et al. 2001)) substrates describe their fucose-binding lectins as players in animal recognition and mycorrhiza formation (Lebreton et al. 2021). Lectins are widely distributed oligosaccharide-binding proteins with diverse specificities. They take part in cell–cell interactions and, especially in plants, are believed to be involved in defense against pathogens. In fungi, fucose-binding lectins play a role in *Aspergillus fumigatus* human infection (Sakai et al. 2019), nematode infection by *Arthrobotrys oligospora* (Tian et al. 2020) and antifungal activity of *Aleuria aurantia* L-fucose-specific lectin against *Mucor racemosum* (Amano et al. 2012).

Fucose was long known as a constituent of the cell wall of *Mucoromycota* (Bartnicki-Garcia 1968), but the structural underpinnings were described only recently in *Mucorales (*Lecointe et al. 2019; Mélida et al. 2015*)*. Fucose constitutes up to 25% of all carbohydrates in the cell wall of two analyzed *Umbelopsis* strains (Muszewska et al. 2021). Remaining subphyla of Mucoromycota, namely *Glomeromycotina* and *Mortierellomycotina*, as well as non-Mucorales orders within Mucoromycotina: *Umbelopsidales* and *Endogonales* remain understudied. Studies have also shown fucose to comprise from 9 to 25% of the EPS layer in several species of *Rhizopus* and *Mucor* (Lecointe et al. 2019; De Ruiter et al. 1992). Furthermore, *Mucorales* secrete exopolysaccharides with significant amounts of L-fucose (Miyazaki and Irino 1972).

Fucosylated proteins were reported in fungi only once (Grass et al. 2011). Enzymes responsible for fucosylation in fungi remain elusive. Although α-1,6-fucose residues in fungal glycans were confirmed, until today there was no evidence of fungal enzymes responsible for this linkage (Martínez-Duncker et al. 2014).

In previous studies, we identified protein *O*-α-fucosyltransferase as one of the protein families forming fungal adaptasome, the dynamically evolving part of protein-coding set of genes(Orłowska and Muszewska 2022) and confirmed a high content of fucose in *Umbelopsis* cell wall (Muszewska et al. 2021). These surprising discoveries and the lack of knowledge on fucose metabolism in fungi led us to ask questions about the occurrence and distribution of proteins associated with fucose in the fungal tree of life (FToL). Hence, we provide a comprehensive screening of proteins involved in fucose metabolism across the fungal kingdom and propose a hypothetical metabolic map.

## METHODS

The starting point for this study was to create the initial list of all possible proteins associated with fucose metabolism across the whole tree of life. We searched the following databases using key words “fucose,” “fucosy,” “fucos”: BRENDA (Chang et al. 2021), UniProt (UniProt Consortium 2021), MetaCyc (Caspi et al. 2016), Pfam (Mistry et al. 2021), TCDB (Saier et al. 2021), UniLectins (Bonnardel et al. 2019). As a result, 75 protein families were identified (38 enzymes, 18 transporters and 19 lectins). For each record from the initial list, we picked reference sequences that were used as a query for searching against the NCBI NR protein sequence database (Sayers et al. 2022) (Additional file 3: Table S1). Reference sequences were chosen based on UniProt annotation score. Prepared queries were used to perform blastp v. 2.8.1 + (Altschul et al. 1990) against NCBI NR database last updated December, 2nd, 2021 limited to fungal taxa with e-value <  = 1e−5. Fasta sequences for obtained accession numbers were downloaded using NCBI Entrez (Sayers et al. 2022). For each set of proteins, we performed clustering in CLANS with p-value thresholds of 1e−5, 1e−10, 1e−15 and 1e−20, expulsive force exponent set to 2 and remaining parameters set as default values (Frickey and Lupas 2004). To eliminate false positives, we analyzed resulting clusters in terms of putative protein function based on domain architecture predicted by PfamScan with default parameters against Pfam database v. 33 and v. 35 (Mistry et al. 2007) and InterProScan 5.60–92.0 (Jones et al. 2014). Fifteen enzymes, 2 groups of transporters and 9 lectins were confirmed to be present in fungal protein sets derived from genomic sequencing (proteomes). Logos representing sequence alignment were prepared in WebLogo 3.7.12 (Crooks et al. 2004). Metabolic maps were built with Inkscape (Inkscape Project 2020) based on pathways stored in MetaCyc database (Caspi et al. 2016). Cellular localization and the presence of signal peptides were predicted by using WoLF PSORT v. 0.2 (organism type—fungi, other parameters set with defaults) (Horton et al. 2007) and SignalP v 5.0 (organism type—eukaryotes, other parameters set with defaults) (Almagro Armenteros et al. 2019). Alignments were calculated using MAFFT v7.490 (–localpair) (Katoh et al. 2002), and trimmed with TrimAl 1.2rev59 (-automated1) (Capella-Gutiérrez, et al. 2009). Phylogenetic trees were constructed with IQTree 2.0.7 (-m MFP -alrt 0 -t RANDOM -nt AUTO) (Minh et al. 2020). Phylogenetic trees included as an outgroup fucosyltransferases identified with the same query list Additional file 3: Table S1) in *Capsaspora owczarzaki* (GCF_000151315.2) (Suga et al. 2013), *Salpingoeca arctica* (GCF_001186125.1) (Ruiz-Trillo et al. 2007), *Fonticula alba* (GCF_000388065.1) (Ruiz-Trillo et al. 2007), *Salpingoeca rosetta* (GCF_000188695.1) (Fairclough et al. 2013), *Monosiga brevicollis* (GCF_000002865.3) (King et al. 2008), Mus musculus (GCF_000001635.27) (Church et al. 2011), Drosophila melanogaster (GCF_000001215.4) (Matthews et al. 2015) and Homo sapiens (GCF_000001405.40_GRCh38.p14) (Lander et al. 2001).

To verify the binding affinity of identified fungal Fringe homologs (*O*-fucosylpeptide 3-beta-*N*-acetylglucosaminyltransferase), we clustered them with reference sequences (Uniprot: O09008 and Q96EU7) using CLANS (same parameters as above) (Frickey and Lupas 2004). We performed structure prediction with ColabFold v 1.4.0 (default parameters) (Mirdita et al. 2022; Jumper et al. 2021). Structures of proteins and ligands were prepared for molecular docking with Open Babel 3.0.1 (O’Boyle et al. 2011). Docking was performed with AutoDock Vina 1.2.0 (Eberhardt et al. 2021) via PyRx 0.9.2 (Dallakyan and Olson 2015) with default parameters. As ligands, we used α-L-fucosyl-L-threonyl residue and *O*-(3-*O*-D-galactosyl-*N*-acetyl-β-D-galactosaminyl)-L-serine.

In order to verify the expression of our predicted proteins, we made use of publicly available transcriptomic datasets of different species of early-diverging fungi (EDFs). The data found were whole transcriptome datasets obtained from pure culture as well from stress conditions. They were collected from the ENA server as fastq files. They were quality checked using FastQC (v0.11.8) (Andrews and Others 2010), followed by trimming of adapters using fastp (v0.19.6) (S. Chen et al. 2018) with default parameters, respectively. The reference genomes of the EDF species were retrieved from Ensembl Fungi (release 53) and aligned with the trimmed reads using Hisat2 (Sirén, Välimäki and Mäkinen 2014). The SAM files obtained from alignment were compressed into binary file format using samtools (v1.10). Finally, the aligned reads were counted for their expression using featureCounts (v1.6.3) (Liao et al. 2014) taking their respective annotations in the form of GTF files from Ensembl (Yates et al. 2022).

The TPM (transcripts per million reads) values and coefficient of variation of TPM were calculated to determine protein expression from whole transcriptome data (Stanton et al. 2017), while differential gene expression was carried out using DESeq2 R package (Love et al. 2014) for protein-coding gene expression from stress transcriptomic datasets. The reads were then mapped with fucose-related protein-coding genes. The log2 fold change criteria of [downregulation < 0 > upregulation] were used to determine their expression profiles during stress and coefficient of variation was used as a determinant of expression from whole transcriptome analysis.

## RESULTS

**Fig. 1:**
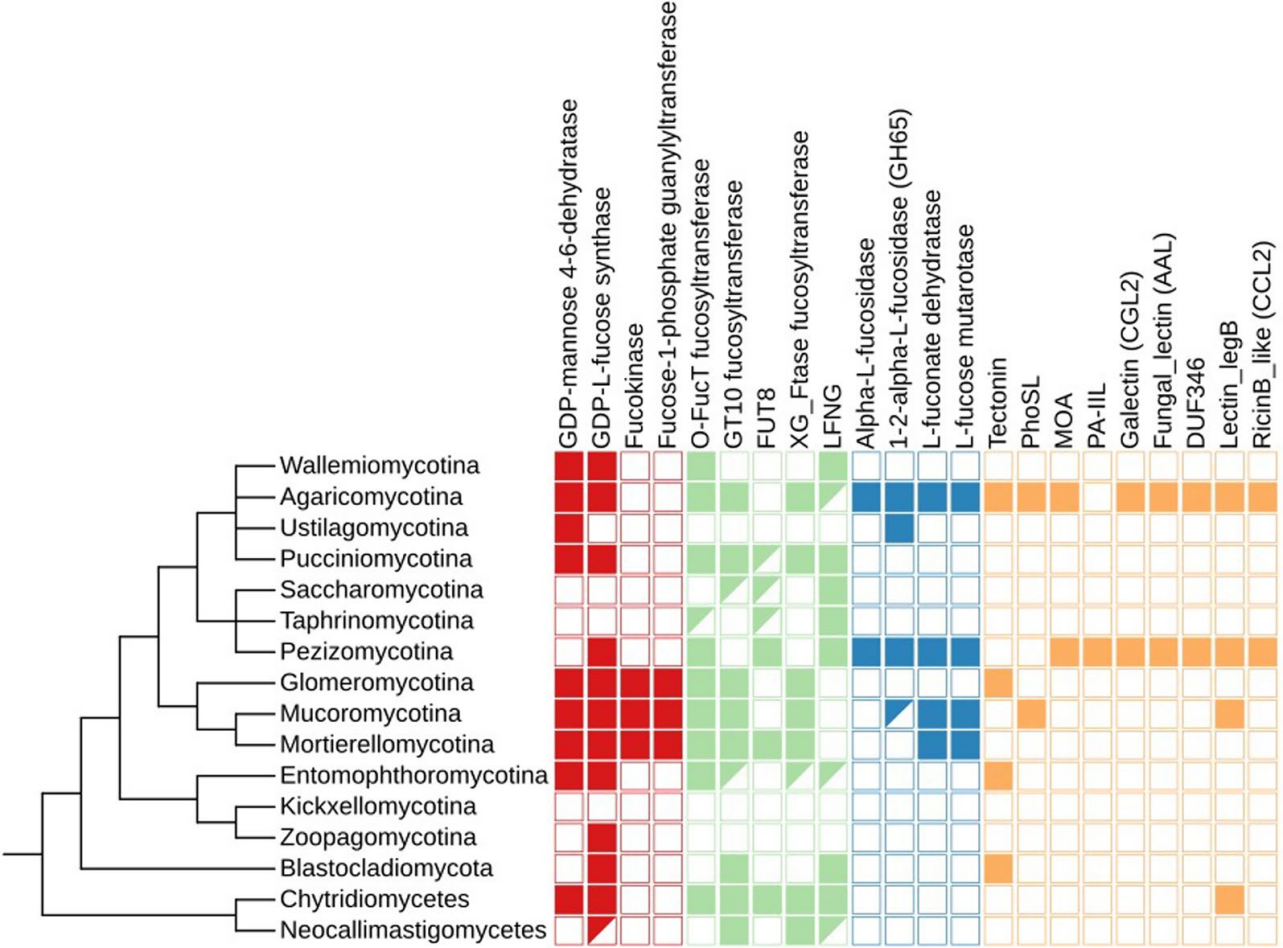
Distribution of fucose-related proteins among the fungal tree of life. Enzymes involved in GDP-L-fucose synthesis are denoted with red squares, transferases with green, enzymes involved in degradation of complex compounds with blue and lectins with orange. The partially colored squares denote single occurrences. In the case of *Basidiobolus* spp. and *Endogonales*, the distribution of proteins is often outstanding from the rest of their taxa (see Additional file 1). The schematic tree is based on (Davis et al. 2019) and (Spatafora et al. 2016)

To construct the fungal fucose metabolic map and to uncover possible fucose-related functions in fungal cells, we analyzed known eukaryotic and prokaryotic pathways. We used 75 protein families—38 enzymes, 18 transporters and 19 lectins from the whole tree of life as references (Additional file 3: Table S1, Table 1). Out of the aforementioned 15 enzymes, 2 groups of transporters and 9 lectins were confirmed to be present in fungal protein sequence sets (Fig. 1). None of the bacterial fucose-related proteins had fungal homologs. Most of the identified fungal homologs belonged to protein families previously described in metazoa. Only a few were reported from fungi, for instance, lectins, fucose synthase and GT10 fucosyltransferases. Seven main functions in which the studied proteins are involved can be distinguished: 1. Nutrition; 2. Fucose biosynthesis; 3. Fucose recycling; 4. Fucosylation; 5. Fucose degradation; 6. Fucose binding (lectins); 7. Transport of fucose. Here, we navigate the fucose-related metabolic map based on the protein functions.

**Table 1:** Fungal enzymes involved in fucose metabolism

EC	Recommended Name	Pfam id	Pfam name	Function	Uniprot reference
1.1.1.271	GDP-L-fucose synthase	PF01370	Epimerase	GDP-L-fucose synthesis (de novo)	Q13630
4.2.1.47	GDP-mannose 4,6-dehydratase	PF16363	GDP_Man_Dehyd	GDP-L-fucose synthesis (de novo)	O60547
2.4.1.152 2.4.1.214 2.4.1.65	L-fucosyltransferases	PF00852, PF17039	Glyco_transf_10, Glyco_tran_10_N	Protein/lipids modification	P22083 Q11130 P21217
2.4.1.-	Fucosyltransferase	PF03254	XG_FTase	Polysaccharide modification	Q9SWH5
2.4.1.221	peptide-*O*- fucosyltransferase	PF10250	O-FucT	Protein modification	Q9Y2G5
2.4.1.68	glycoprotein 6-alpha- L-fucosyltransferase	PF14604, PF19745	SH3_9, FUT8_N_cat	Protein modification	Q9BYC5
2.4.1.222	*O*-fucosylpeptide 3-beta-N-acetylglucosaminyltransferase	PF02434	Fringe	Fucosylated protein modification	Q8NES3
2.7.1.52	fucokinase	PF07959,PF08544, PF00288	Fucokinase, GHMP_kinases_C, GHMP_kinases_N	GDP-L-fucose synthesis (salvage)	Q8N0W3
2.7.7.30	fucose-1-phosphate guanylyltransferase	PF07959	Fucokinase	GDP-L-fucose synthesis (salvage), fucose recycling	O14772
3.2.1.51	alpha-L-fucosidase	PF01120, PF16757	Alpha_L_fucos, Fucosidase_C	Fucose degradation	Q9BTY2
3.2.1.63	1,2-alpha-L-fucosidase	PF14498	Glyco_hyd_65N_2	Fucose polysaccharide degradation	Q6JV24
4.2.1.68	L-fuconate dehydratase	PF13378, PF02746	MR_MLE_C, MR_MLE_N	Fucose degradation	Q8P3K2
5.1.3.29	L-fucose mutarotase	PF05025	RbsD_FucU	Fucose degradation	A2VDF0

### Nutrition

Fucose as a simple hexose can be an easy source of energy. The use of fucose as a nutrient is possible due to degradation of complex compounds by ɑ-L-fucosidase (EC: 3.2.1.51 and 3.2.1.63) (Paper et al. 2013). α-L-fucosidases belong to either CAZy glycoside hydrolases family GH29 and are characterized by domains Alpha_L_fucos (PF01120) and Fucosidase_C (PF16757) or they belong to family GH95 and have a Glyco_hyd_65N_2 (PF14498) domain. Fucosidases from family GH29 act on fucosyl linkages α-1,2/3/4/6, while GH95 homologs act only on α-1,2 linkage (Paper et al. 2013). Identified proteins from both families are predicted to have extracellular localization.

The ability to treat fucose as a nutrient inferred from the presence of ɑ-L-fucosidase coding genes seems to be limited to Dikarya. We found homologs of this enzyme only in the representatives of *Agaricomycotina*, *Ustilagomycotina* (*Basidiomycota*) and *Pezizomycotina* (*Ascomycota*). Notably many of the taxa encoding ɑ-L-fucosidase genes rely on plant organic matter either as pathogens of saprotrophs or belong to lineages evolutionarily linked to plant hosts. We found only one homolog of this family in *Mucoromycotina* which suggests the possibility of contamination.

### Fucose biosynthesis and recycling

**Fig. 2:**
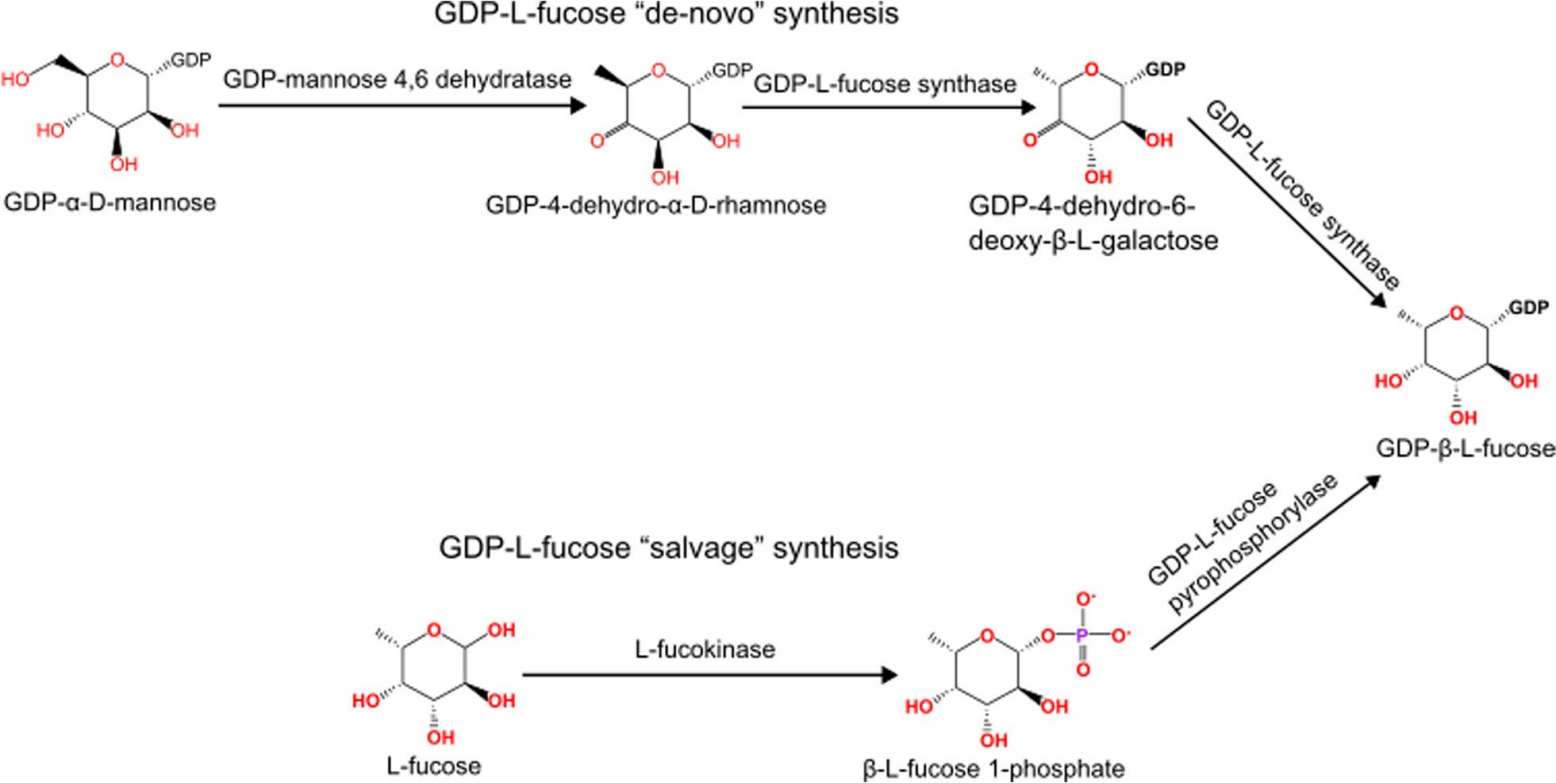
GDP-L-Fucose synthesis pathways. Both *de-novo* and salvage pathways are present in Fungi

Fucose can be a building block of complex carbohydrates and be added to diverse molecules. As such, it needs to be acquired or synthesized in the fungal cell. GDP-L-fucose is an activated nucleotide form of L-fucose and is required for fucosylation carried out by fucosyltransferases. GDP-L-fucose may be synthesized in two ways: via* de-novo* and salvage pathways (Fig. 2). Components of the salvage pathway (also of the fucose recycling process)—fucokinase FUK (EC: 2.7.1.52) and fucose-1-phosphate guanylyltransferase GFPP (EC: 2.7.7.30) are present only in *Mucoromycota*—we found only single homologs of FUK and GFPP outside this taxon in some chytrids. The enzymes involved in the de novo synthesis pathway (GDP-mannose 4,6-dehydratase (EC: 4.2.1.47) and GDP-L-fucose synthase (EC: 1.1.1.271)) are present in all sequenced *Mortierellomycotina* but not limited to this taxon. Representatives of Dikarya, *Zoopagomycota*, *Chytridiomycota* and *Blastocladiomycota* can probably synthesize GDP-L-fucose de novo. However, the conservation of this pathway is incomplete. Several *Ascomycota* (*Pezizomycotina,* *n* = 11*)* only have the second enzyme in the pathway the GDP-L-fucose synthase, whereas *Ustilagomycotina* have GDP-mannose 4,6-dehydratase alone.

### Fucosylation

**Fig. 3:**
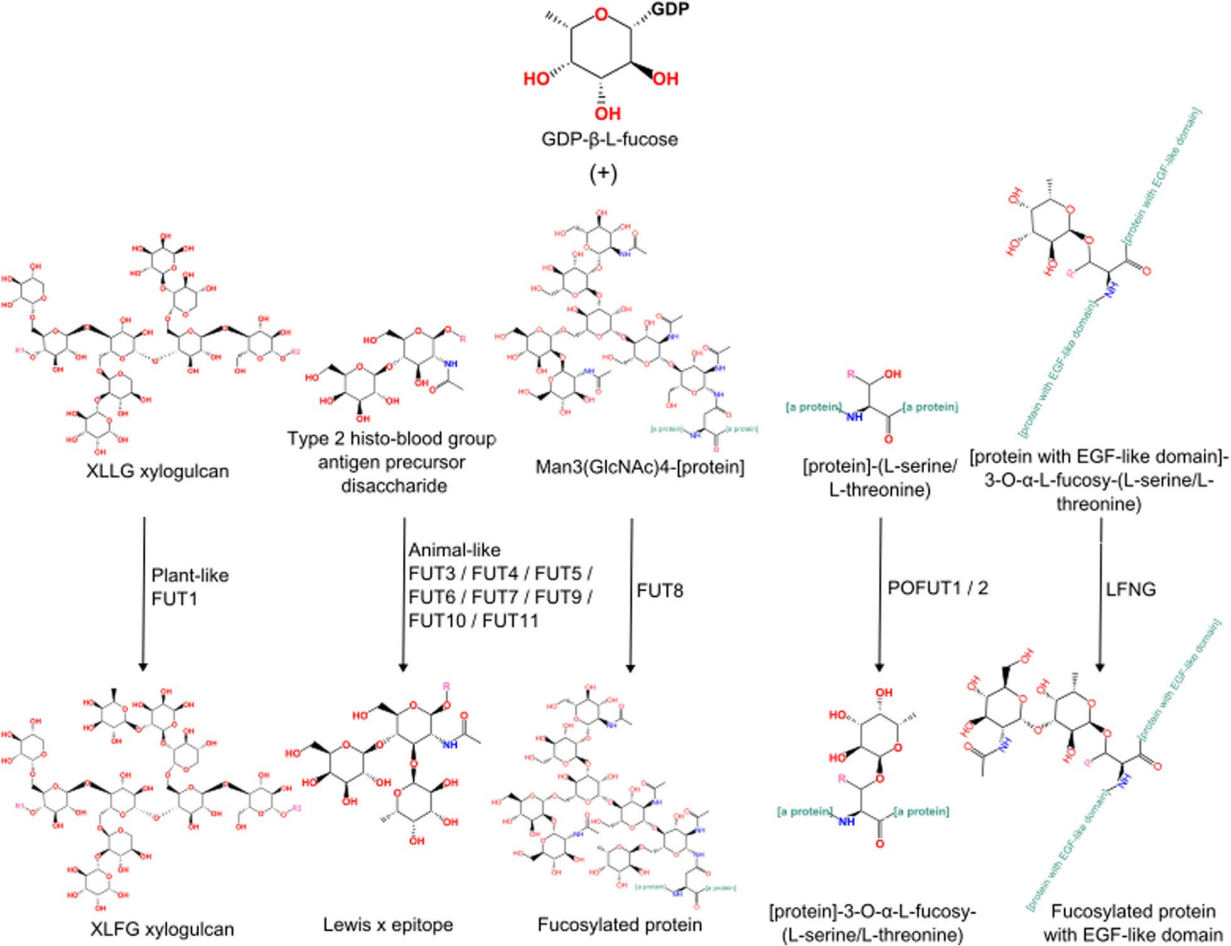
Fucose-related modification pathways in *Fungi*

**Fig. 4:**
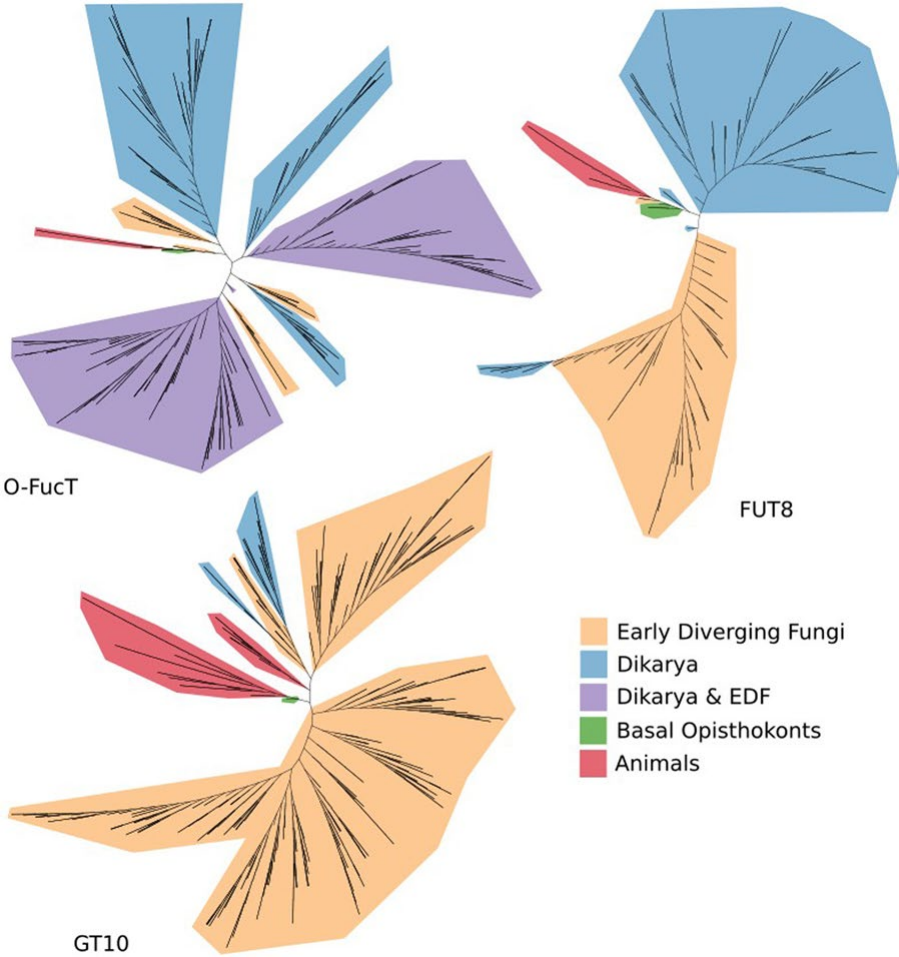
Unrooted maximum likelihood phylogenetic trees of three fucosyltransferases families (Additional file 2)

Fungi possess five families of enzymes involved in fucose-related modification of proteins, lipids and polysaccharides (Fig. 3). These fucosyltransferases are homologous to animal transferases (Fig. 4). These enzymes belong to two Pfam clans GT-A and GT-B, both possessing a Rossman fold and grouping diverse glycosyltransferases. *O*-fucosylpeptide 3-beta-*N*-acetylglucosaminyltransferase (LFNG) (EC: 2.4.1.222) defined by Fringe domain (PF02434) is a transferase belonging to Pfam clan GT-A (CL0110). This protein is not able to fucosylate, but is interesting for this work as it operates on fucosyl peptides.

Fringe proteins act on fucosylated and galactosylated peptides (Ju and Cummings 2002). These two groups are highly similar in terms of sequence and structure. We were unable to recognize homologs of Fringe-domain containing proteins that act exclusively on fucosyl peptides (neither based on sequence and structure nor by molecular docking of simplified ligands, see Additional file 1: for clustering results).

**Fig. 5:**
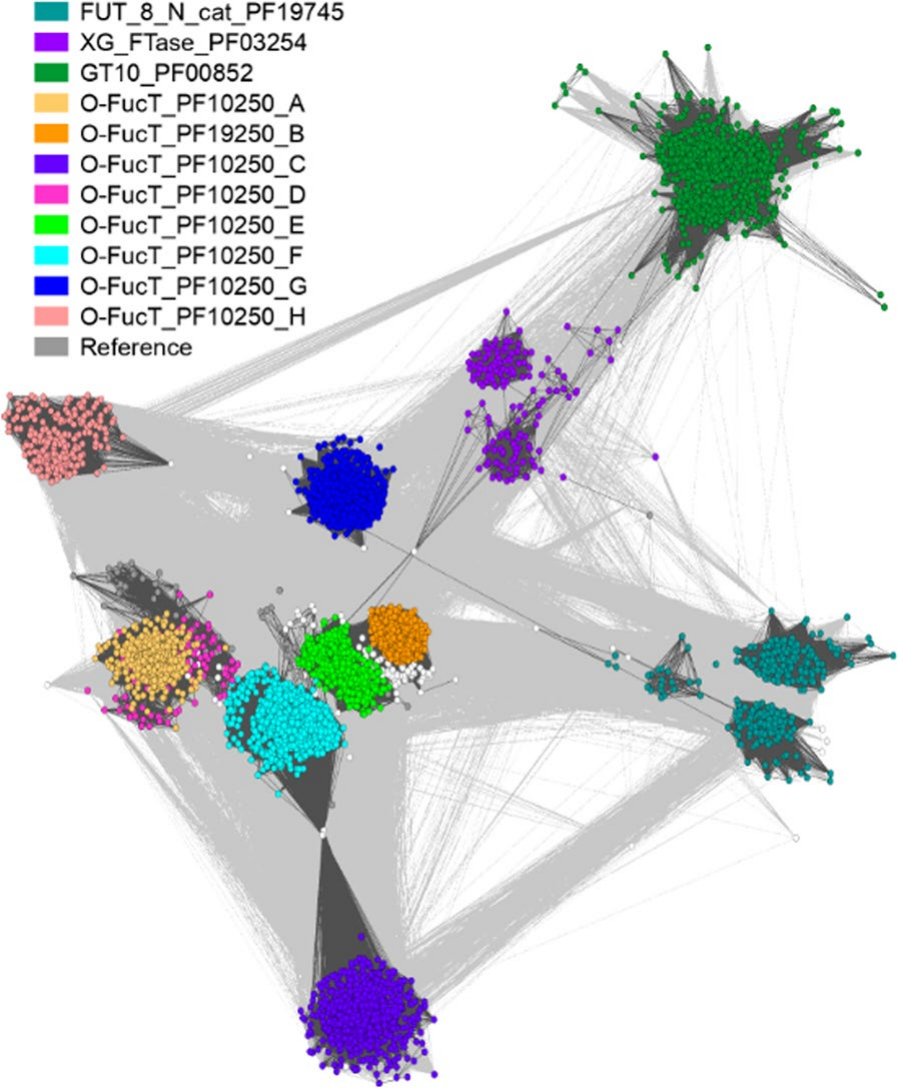
GT-B clan (CL0113) fucosyltransferases present in fungi with new O-FucT subfamilies

**Fig. 6:**
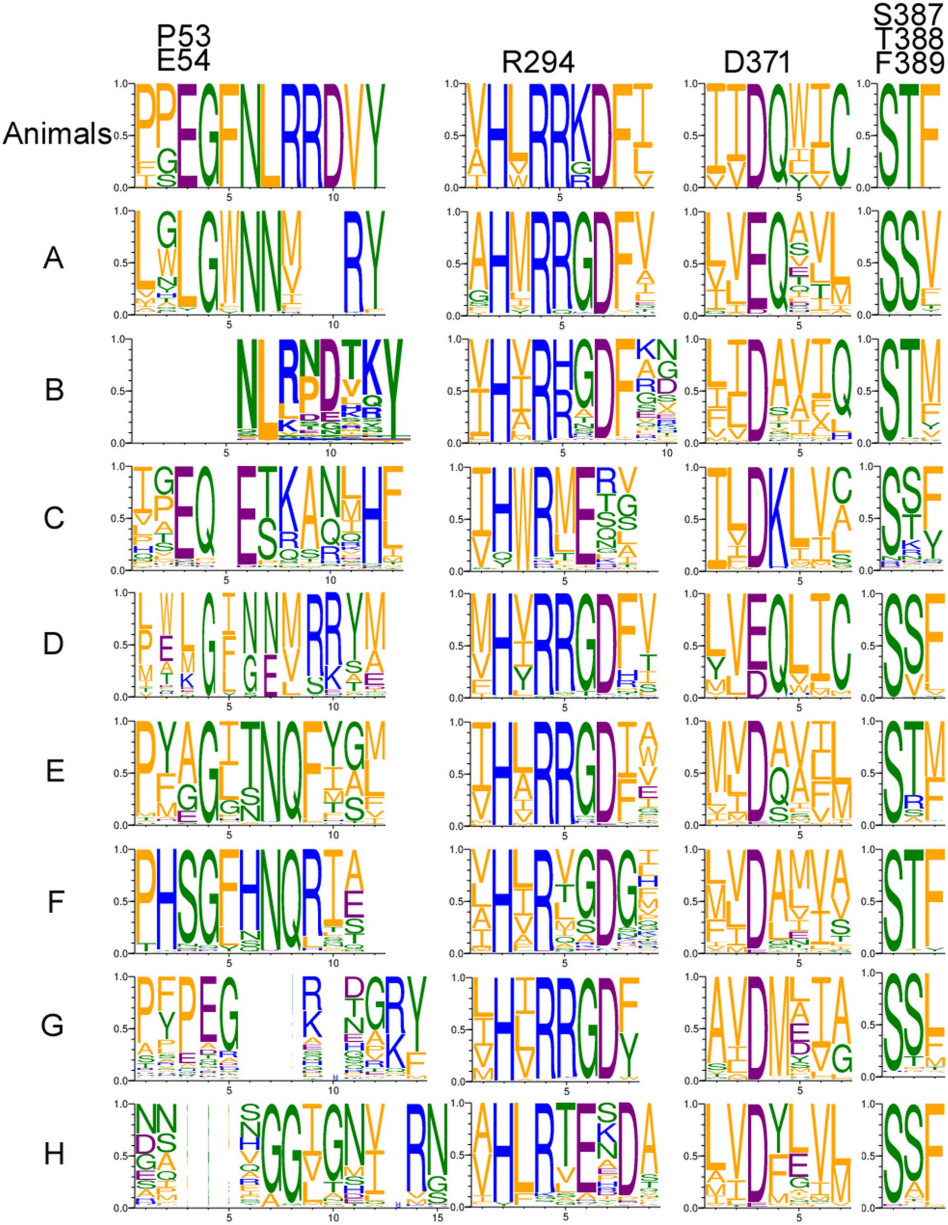
Sequence logos of active site and GDP-fucose-binding motifs for selected subfamilies (see description in the text above). Animal O-FuT is shown as a reference (Chen et al. 2012). Letters correspond to clusters pictured in Fig. 5 (clans)

The remaining four enzymes are representatives of the GT-B clan (CL0113) (Fig. 5). Homologs of CAZY glycosyl transferase 10 family (GT10, EC: 2.4.1.152 / 2.4.1.214 / 2.4.1.65) have a very similar distribution to proteins with a XG_FTase (PF03254) domain. Both families are present in *Pucciniomycotina, Agaricomycotina, Mucoromycota* and *Chytridiomycota. Blastocladiomycota* fungi seem to have only GT10 homologs in terms of fucosyltransferases in general. Interestingly, *Basidiobolus* is the only *Zoopagomycota* lineage encoding a rich repertoire of fucosyltransferase genes (GT10, XG_FTase and O-FucT). Proteins with O-FucT domain (PF10250) (peptide-*O*-fucosyltransferase, EC: 2.4.1.221) make up the most diverse group of fucosyltransferases. We recognize eight subfamilies of these transferases mapping on the Pfam O-FucT domain but with distinguishable sequence motifs and taxonomic distribution (Figs. 5 and 6). Two subfamilies are plant-specific and the remaining ones are fungal subfamilies with the highest occurrence in *Agaricomycotina*, *Pucciniomycotina* and *Mortierellomycotina*. Representatives of O-Fuct are present in the earliest fungal lineages including *Rozella* and *Olpidium*. Still, they show a repeated loss of this trait in yeast-forming fungi (*Ustilagomycotina*, *Saccharomycotina*, and just a single copy in *Taphrinomycotina*). The characteristic sequence motifs of each new O-FucT subfamily corresponding to conserved sequences of POFUTs fucosyltransferases (Chen et al. 2012) are summarized with alignment logos (Fig. 6). Almost all novel subfamilies have well-preserved key POFUTs residues; we analyzed the conservation of catalytic amino acids residues—E54 and R294, as well as conservation of amino acids critical for GDP-fucose binding—P53, D371, S387, T388 and F389 (Chen et al. 2012). R294 residue is well preserved among all identified POFUTs. In the case of E54, this residue is conserved only in C and G, but the surroundings keep a similar, aromatic character in most POFUTs—in A, B, F. P53 have a very similar distribution. In the case of D and E, we can observe well-conserved G55 residue, but without following aromatic residues. D371 and S387 are strongly conserved among all groups. T388 and F389 are not very well conserved but are substituted by amino acids with similar properties.

Subfamily G encompasses characterized fucosyltransferases involved in strobilurin polyketide modification (Nofiani et al. 2018), cell-cycle regulation coupled with cell signaling and differentiation. O-FucT subfamilies present in *Dikarya* A and B are surrounded by secondary and cellular metabolism genes acting as a regulator of autophagy with its role in the formation of autosomes and in vacuolar protein sorting. The subfamilies E and F are also involved in secondary metabolism since the subfamily E genes co-occur with polyketide synthetase, ketopantoate reductase as well as MFS1 class transporters found ubiquitously in SMC. Genes encoding fucosyltransferases from subfamilies A and B occur mostly in a housekeeping neighborhood related to transcription, DNA repair and lipid metabolism. Subfamily C present mostly in EDFs may have an intracellular role linked to dolichol metabolism due to the occurrence of decaprenyl diphosphate synthase-like and undecaprenyl diphosphate synthase domains.

We did not find fungal homologs of animal FUT1 and FUT2 transferases, which belong to the CAZY GT11 family. FUT1,2 catalyzes the transfer of fucose to the terminal galactose through ɑ-1,2 linkage (Kudo and Narimatsu 2008).

Enzymes with FUT8_N_cat domain (PF19745) (glycoprotein 6-alpha-L-fucosyltransferase, EC: 2.4.1.68) are mostly present in *Mortierellomycotina* and *Chytridiomycotina.* In *Dikarya*, they are present in *Pezizomycotina* (only a few homologs), *Pucciniomycotina, Taphrinomycotina* and *Saccharomycotina.* The *Dikarya* homologs are mostly present in entomopathogenic fungi and form a separate clade in sequence clustering (Fig. 5). *Mortierellomycotina* sequences group into a clade distinct from the *Dikarya*.

RNA-Seq data analysis identified the expression of four O-FucT subfamilies. While *Mucoromycotina* and *Glomeromycotina* expressed protein-coding genes from subfamilies C and E, *Mortierellomycotina* expressed subfamilies C, F and G from the whole transcriptome data. The analysis of stress response data displayed up and downregulation of fucose protein-coding genes in *Glomeromycotina*. All of the genes expressed in *Mucoromycotina* were upregulated. We did not find any publicly available transcriptomic stress experiments in organisms belonging to Mortierellomycotina.

### Fucose degradation

**Fig. 7:**
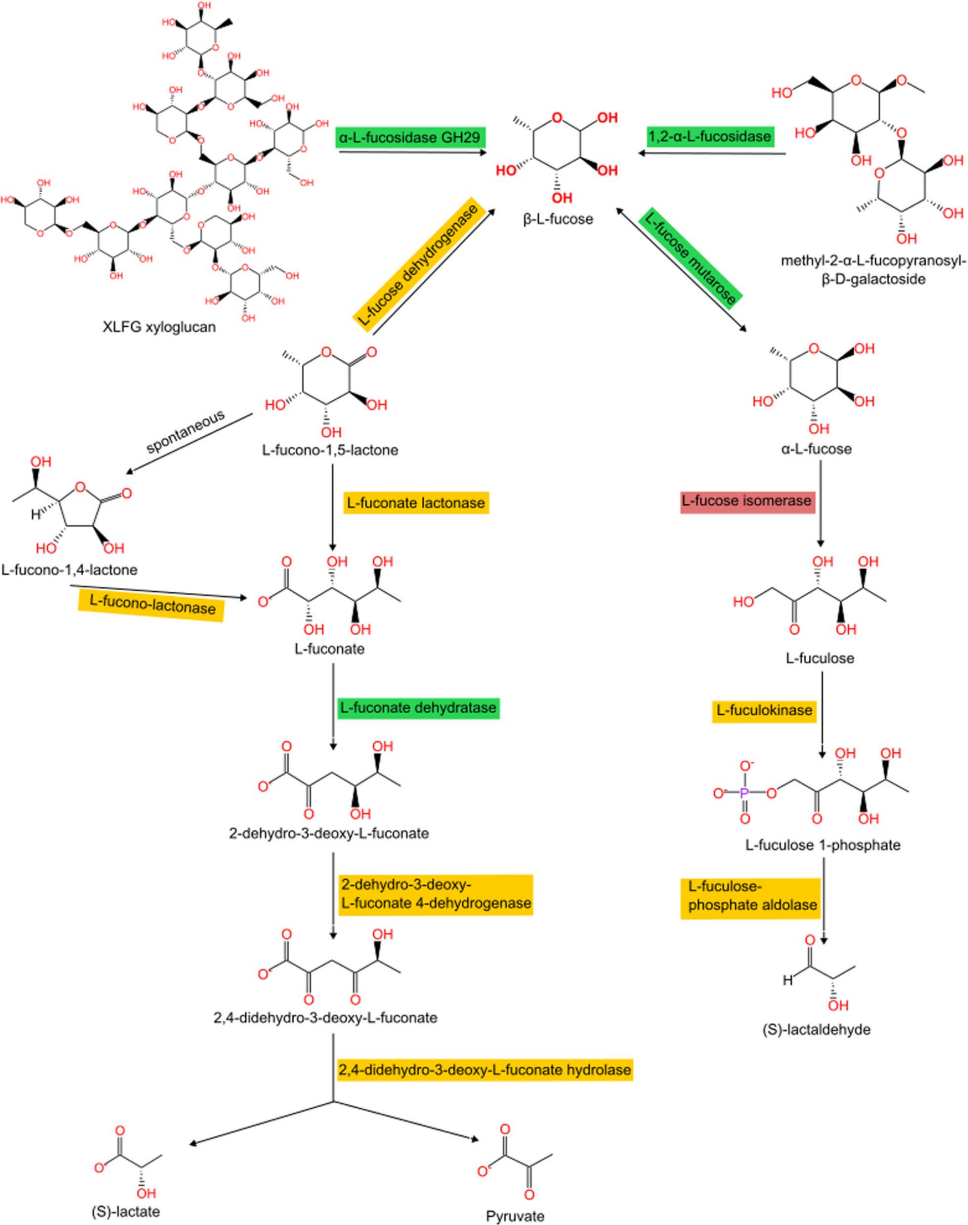
Catabolic pathways of fucose-containing compounds. Homologs of enzymes defined in MetaCyc pathways present in Fungi are marked in green; red-marked enzymes are not present in Fungi; products of reactions marked in yellow have been detected in Fungi but corresponding protein and gene sequences are not known or reported enzymes show marginal specificity (e.g., AA12 as fucose dehydrogenase and lactonase)

In fungi—in addition to alpha-L-fucosidase GH29 involved in nutrition—three enzymes are responsible for the degradation of fucose and fucose-containing polysaccharides (Fig. 7). The most numerous is the family of L-fuconate dehydratase (EC: 4.2.1.68). These proteins are built from the domains MR_MLE_C (PF13378) and MR_MLE_N (PF02746). They are present in *Agaricomycotina, Pezizomycotina, Mortierellomycotina* and *Mucoromycotina.* The same taxonomic distribution was observed for L-fucose mutarotase (EC: 5.1.3.29), defined by the RbsD_FucU (PF05025) domain. 1,2-alpha-L-fucosidase (EC: 3.2.1.63) homologs play a role in the degradation of polysaccharides by cleaving fucose residue. They have Glyco_hyd_65N_2 (PF14498) domain and occur in *Ustilagomycotina, Agaricomycotina, Pezizomycotina.* A single sequence was also found in *Umbelopsis vinacea.* The relatively narrow distribution of both enzymes opens the question of how other fucose-containing lineages degrade this substrate.

The MetaCyc database describes two L-fucose degradation pathways (named I and II). Fungi probably do not have L-fucose degradation pathway I as we did not detect L-fucose isomerase. We found only two homologs of this enzyme in *Hortaea werneckii* and *Mortierella* sp. GBA39. These sequences show extreme similarity to bacterial sequences from the *Paenibacillus* genus that point to contamination of fungal genomic sequences. The function of other enzymes of pathway—L-fuculokinase and L-fuculose-phosphate aldolase—may be handled by fungal homologs with the same domain architectures described as xylulose kinase and unannotated aldolases, but the question remains if these enzymes have appropriate substrate specificity. In the case of degradation pathway II, we do not find homologs of specific enzymes described as pathways components in the MetaCyc database, but we speculate that the function of these enzymes may be handled by other aldolases, hydrolases and dehydrogenases. For example, TrAA12 dehydrogenase from *Trichoderma reesei* shows weak activity against uncommon carbohydrates including L-fucose (Turbe-Doan et al. 2019).

### Fucose binding (lectins)

We identified nine families of proteins that can bind compounds with fucose or bind fucose directly. Fucose-binding lectins are most abundant in *Dikarya*, particularly in *Pezizomycotina* and *Agaricomycotina*. We found homologs of previously described fungal lectins like *Aleuria aurantia* lectin (AAL) group (Fungal_lectin (PF07938)) (Wimmerova et al. 2003), galectins (Gal-bind_lectin (PF00337)), *e.g., Coprinus cinereus* protein CGL2 (Cooper et al. 1997), *Pholiota squarrosa* lectin (PhoSL) (also called RSL when from *Rhizopus stolonifer (*Oda et al. 2003*)*), tectonins (PF19193), *e.g.,* LbTec2 from *Laccaria bicolor (*Sommer et al. 2018*)*, Agglutinin_C (PF14200) and RicinB_lectin_2 (PF14200) domains containing proteins like MOA lectins (*M. oreades* agglutinin) (Grahn et al. 2009) and RicinB_like family (CCL2 protein from *C. cinerea*. CCL2 (Schubert et al. 2012).

The other lectins found are known to date from other organisms but were not described in fungi—DUF346 (PF03984) lectins, PA-IIL (PF07472) and Lectin_legB (PF00139). Outside *Dikarya*, we found lectins in *Glomeromycotina* and *Blastocladiomycota* (Tectonin), *Mucoromycotina* (PhoSL and Lectin_legB) and *Chytridiomycota* (Lectin_legB).

### Transport of fucose

TCDB classification was used to determine candidates for fucose transporters. All identified fungal proteins belong to 2.A.7 family, subfamilies 2.A.7.10.4 and 2.A.7.16. Fungal 2.A.7.10.4 family transporters form three groups, each with a different taxonomic distribution. The first group consists of *Basidiomycota* and EDF sequences, the second group includes only *Ascomycota* sequences and the third group is *Basidiomycota* specific. In the case of 2.A.7.16 fungal transporters, we also observed three groups. Two of them are specific for EDF (one consists only of *Mortierellomycotina* and *Glomeromycotina* homologs) and one group for both EDF and Dikarya sequences.

### Fucose-related traits not confirmed in fungi

L-fucose is the main component of fucoidan—a complex polymer with sulfation and acetylation modifications produced mostly by brown algae (Li et al. 2008). We did not find homologs of enzymes capable of synthesizing fucosyl polymers and in consequence have no intermediate evidence for their existence in fungi. For instance, we have not found homologs of enzymes involved in fucose polysaccharide synthesis like TDP-fucosamine acetyltransferase, fucoidan-2-sulfatase and UDP-*N*-acetyl-L-fucosamine synthase. Fucose polymer degradation, if existent, has to be performed without homologs of bacterial fucoidanases. We did not identify any of the prokaryotic components of the fucose metabolism. Suppose fungi are able to synthesize secondary metabolites with fucosyl residues. In that case, the synthesis has to be independent of dTDP-fucopyranose mutase, aclacinomycin-T 2-deoxy-L-fucose transferase and dTDP-alpha-D-fucopyranose:NAD(P) + oxidoreductase. Bacterial antigen biosynthesis was also missing based on the absence of TDP-*N*-acetylfucosamine: lipid II *N*-acetylfucosaminyltransferase and GDP-beta-L-fucose: beta-D-Gal-(1- > 3)-alpha-D-GalNAc-(1- > 3)-alpha-D-GalNAc-diphospho-ditrans, octa-cis-undecaprenol alpha-1,2-fucosyltransferase.

## DISCUSSION

Our aim in this work was to provide a generalized picture of fucose usage and metabolism on the fungal tree of life. To design the metabolic map, we have compared existing prokaryotic and eukaryotic proteins depending on or using fucose residues in any form. We have then detected their presence in diverse fungal lineages and reconstructed metabolic maps for the main fungal lineages taking into account an ecological and biochemical perspective. Most of the identified protein families are homologs of animal fucose-related proteins and none of the bacterial protein families was found in fungi. This led us to predict different patterns of fucose usage in EDF compared to *Dikarya*. The former seems to predominantly use fucose either as a building block for polymers or as a residue for macromolecule modification. The latter detects fucose in their hosts with lectins and possesses a set of putative enzymates that allows to use it as an energy source. Noteworthy, yeast-forming lineages of both *Ascomycota* (*Saccharomycotina*) and *Basidiomycota* (*Ustilagomycotina*) have lost most of the fucose-related protein-coding genes. Overall the evolutionary history of fucose metabolism in Fungi seems to be shaped by consecutive and parallel losses of its components, leading to its simplification or even complete loss. The overall fucose metabolic capabilities of fungi and animals remain similar. One of the fucosyltransferase families GT11 is apparently lost in all *Fungi*.

Most of the fucose-related proteins form a coherent group in terms of sequence diversity, confirming a common origin predating the radiation of animals and fungi. Despite the ubiquity of endohyphal bacteria among fungi, and documented co-evolution with some *Mucoromycota* taxa, we observed no transfer of fucose metabolic genes to fungal genomes. We have, however, detected a burst of diversity in the O-FucT fucosylase family with changes both in the core sequence motifs and occurrence in fungal lineages.

The complexity of the fucose map genes varies greatly between fungal lineages, with *Mortierellomycotina* possessing the most complete map among studied taxa. They are the only lineage to encode both fucose synthesis and recycling pathways as well as a wide range of fucosyltransferases including the newly described O-FucT subfamilies. In the case of *Mucoromycotina*, some enzymes (fucokinase, fucose-1-phosphate guanylyltransferase) are limited only to *Endogonales*. On the other hand, all yeast-forming lineages do not use fucose as a building block and hardly ever have fucose-detecting lectins. Most *Dikarya* lineages with a complex fucose map are related to a plant host either as saprotrophs or pathogens.

In our previous work, we showed that two *Umbelopsis* species have up to 25% of fucose in their cell wall (Muszewska et al. 2021). Mucoran is a polysaccharide described in the cell wall of *Mucoromycota*. It is composed of D-glucuronic acid, L-fucose and D-mannose in a molar ratio of 5:2:3 (Bartnicki-Garcia and Lindberg 1972). The composition of the *Umbelopsis* spp. cell wall differs from this ratio. However, we did not find in their genomes homologs of enzymes involved in the biosynthesis of fucose polymers. A question arises about the form of fucose present in *Umbelopsis* cell wall. Some of the remaining fucose may exist as a part of glycuronan—polysaccharide present in *Mucorales* and it is composed of fucose, mannose, galactose and glucuronic acid with a molar ratio of 5:1:1:6 (Lecointe et al. 2019). Mucoran can also be an acceptor of fucose residues (Camacho-Aguero, Balcazar-Orozco, and Flores-Carreo´n 1990). Furthermore, *Mucorales* have also exopolysaccharides (EPS) that contain fucose (De Ruiter et al. 1992) so maybe this is also the case for *Umbelopsis* spp. One might speculate that the detection of high amounts of fucose in the cell wall might be also attributed to the presence of EPS. It is also likely that fucose is localized in both EPS and the cell wall.

### Biosynthesis/recycling

The presence of both GDP-L-fucose synthesis pathways—the de novo and the salvage pathway in *M. alpina*—was reported already in 2010 (Ren et al. 2010; Wang et al. 2016). According to our results, both pathways co-occur only in *Mortierellomycotina.* Our results suggest that the salvage pathway is limited to *Mucoromycota.* As the functional salvage pathway is present in animals (including mammals) (Schneider et al. 2017), we assume that genes encoding components of this pathway were lost in the evolution of fungi outside *Mucoromycota*. An additional argument supporting this thesis is the presence of the salvage pathway in the tree of life before the formation of Opisthokonta—its occurrence was described in *Dictyostelium discoideum (*Gonzalez-Yanes et al. 1989*).*

Enzymes involved in the de novo synthesis pathway are present in all fungal divisions. However, several lineages encode only one of the two enzymes needed which opens the question on the possibility to produce GDP-L-fucose without GDP-mannose 4,6-dehydratase and function replacement by another dehydratase. The presence of both pathways in *Mortierellomycotina* points to a peculiar complexity of fucose metabolism in this fungal subphylum.

### Fucosylation

More than 13 different fucosyltransferases were described in humans (FUTs and POFUTs). They play a role in posttranslational modification of proteins, glycans and lipids (Schneider et al. 2017; Shimizu et al. 2001). We identify five families of fungal fucosyltransferases, all present both in *Dikarya* and EDF. It suggests that fucosylation is an ancient trait common in Opisthokonta. The ancestral set of fucosyltransferases comprises.

CAZY GT10 family (with domains Glyco_transf_10 (PF00852) and Glyco_tran_10_N (PF17039)) homologs of human FUT3-7 and 9–11 involved in the synthesis of Lewis antigens, homologs of plant cell wall-related FUT1 protein (Perrin et al. 1999) characterized by XG_FTase domain (PF03254), homologs of O-FucT called POFUT1 and POFUT2—involved in Notch signaling (Stahl et al. 2008; Sparrow et al. 2006)). The Fungal O-FucT family harbors a few novel subfamilies with derived conservation of the active site motif suggesting a possible change of the protein substrate. This is particularly likely in the absence of EGF motives in fungal hyphae. One might speculate that Dikarya O-FucT subfamilies can be a part of candidate secondary metabolite clusters similar to the strobilurin polyketide. The subfamilies occurring in EDF might be involved in some housekeeping function or signaling based on the genomic neighborhood observations. The revealed complexity of fucosyltransferases is smaller than in Metazoa but greater than expected based on published data. The fucosyltransferase repertoire may work on a wide range of substrates and contribute to novel natural compounds, molecular biology tools or diagnostic markers.

The fungal transferases catalyze the transfer of L-fucose from GDP-L-fucose to the *N*-acetyl glucosamine via linkage ɑ-1,3 (FUT4,6,7,9) or ɑ-1,4 (FUT3,5) (Breton et al. 1998). POFUTs catalyze the reaction by attaching fucose to serine/threonine residues via* O*-glycosidic linkage (C.-I. Chen et al. 2012). We did not find fungal homologs of animal FUT1 and FUT2 transferases, which belong to CAZY GT11 family. FUT1,2 catalyzes the transfer of fucose to the terminal galactose through ɑ-1,2 linkage (Kudo and Narimatsu 2008). But we found homologs of plant FUT1 that operate on the same linkage (Sarria et al. 2001). We also found homologs of FUT8 that add fucose to *N*-glycans via ɑ-1,6 linkage (García-García et al. 2021). The possibility of transfer of *N*-acetyl-α-D-glucosaminyl residue to *O*-fucose-modified peptides/proteins by Fringe homologs opens up several questions regarding what could be the fucosylated peptide/protein and in which biological processes this mechanism would be involved. Taken together, fungi are able to process all kinds of fucosylation known in animals—it is especially true for *Mortierellomycotina*, as fungi from this taxa have homologs of all described transferases from GT-B clan.

### Degradation

In addition to glycoside hydrolase family 29 present in *Dikarya* and involved in nutrition, we found other hydrolases among *Dikarya* and EDF that are involved in the degradation and recycling of fucose within a cell. 1,2-alpha-L-fucosidase (EC: 3.2.1.63) is responsible for the degradation of polysaccharides to L-fucopyranose. This enzyme belongs to the GH95 family and was described before in *Aspergillus niger* (Bahl 1970).

MetaCyc database describes two L-fucose degradation pathways (named I and II). Fungi probably do not have L-fucose degradation pathway I as they lack L-fucose isomerase. We found only two homologs of this enzyme in *Hortaea werneckii* and *Mortierella sp. GBA39*. This sequence shows extreme similarity to bacterial sequences from the *Paenibacillus* genus, which point to contamination of fungal genomic sequences. The function of other enzymes of the pathway—L-fuculokinase and L-fuculose-phosphate aldolase—may be handled by fungal homologs with the same domain architectures described as xylulose kinase (Y. M. Chen et al. 1987) and unannotated aldolases, but the question remains if these enzymes have appropriate substrate specificity.

We hypothesize that fungi may have L-fucose degradation pathway II. However, we did not find homologs of L-fucose dehydrogenase, L-fuconolactonase, 2-dehydro-3-deoxy-L-fuconate-4-dehydrogenase and 2,4-didehydro-3-deoxy-L-fuconate hydrolase, but we speculate that function of these enzymes may be handled by different enzymes that possess the same protein domains. L-fucose dehydrogenase activity may be performed by broad specificity enzymes such as AA12 from *T. reesei* (Turbe-Doan et al. 2019).

### Binding

Lectins are a diverse and widespread group of proteins, capable of binding oligosaccharides in a specific, non-catalytic way (Rini 1995), and are produced by all organisms (Kobayashi and Kawagishi 2014). In Fungi, these proteins are known to play a role in storage, growth, morphogenesis, parasitism, molecular recognition and mating (Kobayashi and Kawagishi 2014). Most of the described fungal lectins were from *Dikarya* (especially fruiting body forming). Fungal lectins are of particular biotechnological interest and numerous studies deciphered their structural properties allowing them to be exploited as a diagnostic tool for cancer or in targeted drug delivery (Varrot et al. 2013).

Lectin LecB (called also PA-IIL, same as domain PF07472) is one of two lectins synthesized by the opportunistic pathogen *Pseudomonas aeruginosa* and are important virulence factors. LecB plays a role, especially in *Pseudomonas* respiratory chronic infection (Tielker et al. 2005). This kind of lectins was never described in fungi before. We observed the occurrence of LecB homologs in a very narrow taxonomic context—they are present in two broad host range *Clavicipitaceae* enthomoparasitic genera, *Metarhizium* and *Hirsutella.* Obtained sequences show similarity to bacterial homologs, *e.g.,* from *Nostoc, Kitasatospora* and *Sorangium,* which may suggest horizontal gene transfer from bacteria. However, as similarity ranks around 40%, and there is no ecological overlap between these bacteria and fungi, the evolutionary origin of these lectins is not clear.

We found homologs of fucose-binding lectin from *Photorhabdus luminescens* (PLL). These proteins are characterized by DUF346 domain (PF03984). *P. luminescens* is a bacteria known for its symbiotic relationship with nematodes from the family Heterorhabditidae and its pathogenicity toward insects (Forst et al. 1997). The biological role of PLL remains unclear. Some sources suggest that PLL has larvicidal activity (Eisemann et al. 1994), while others suggest that the role of PLL is limited to cell recognition and adhesion (Kumar et al. 2016).

Lectin_legB (PF00139) lectins are originally known from plants as legume lectins. *Lotus tetragonolobus* lectin (LTA) is a fucose-specific lectin with described diverse activities (Moreno et al. 2008), *e.g.,* reactivity against blood group O (Konami et al. 1990). Legume lectins play a role in nodulation by *Rhizobium* (Hirsch 1999).

### Transporters

Almost twenty TCDB families of transporters are related to fucose transport. From them, only three subfamilies have homologs in fungi. Proteins from the drug/metabolite transporter (DMT) superfamily 2.A.7 were described among others as Efr and Gfr (ER (endoplasmic reticulum)/Golgi GDP-fucose transporter) in *Drosophila* (Ishikawa et al. 2010) and SLC35C1 in *Homo sapiens (*Ishida and Kawakita 2004*)*. Transport of GDP-fucose into the ER and Golgi is necessary for fucosylation because GDP-fucose is synthesized in the cytosol but fucosylation takes place in the lumen of ER and Golgi (Ishikawa et al. 2010).

Also, ATP-binding cassette (ABC) superfamily transporters (3.A.1) are probably involved in fucose transport in fungi. Proteins from family 3.A.1.2.5 act like multiple sugar transporters with the ability to transport fucose among other sugars. The occurrence of such transporters was described in bacteria (Conners et al. 2005; Kemner et al. 1997). So far fucose transporters were not verified experimentally in fungi.

## CONCLUSION

Here, we present a novel putative metabolic map in fungi with a distinction between non-*Dikarya* and *Dikarya* subtypes of fucose utilization. The former likely depends on fucose as a building and signaling compound while the latter mainly detects it and uses it as a carbon source. We have observed some clear patterns with most of the fucose-related proteins co-occurring within Opisthokonta and no HGT bacterial protein families present in the analyzed fungi. Fungi possess a limited subset of the metazoa fucose metabolic map, however, sufficient to be able to synthesize, degrade, bind, transfer and transport this compound. This ensemble provides a list of hypotheses to be tested experimentally.

### Supplementary Information

**Additional file 1**. CLANS clustering of GT-A clan peptidyltransferases present in Fungi with animal representatives of transferases acting on glucosylated, fucosylated and galactosylated peptides. After clustering of fungal Fringe homologs with 42 animal representatives of both LFNG and C1GLT transferases, we obtained four clusters with 4946 fungal sequences. However, only four fungal sequences (Rozella allomycis RKP19558.1,RKP21212.1, EPZ36320.1 and Fusarium oxysporum EXK84998.1) group together with the human LFNG (Q8NES3) protein sequence. Two major fungal clusters are equally distant from LFNG and C1GLT clans. Basidiobolus meristosporus ORX91553.1 and Batrachochytrium salamandrivorans KAH6567989.1, KAH6579126.1 and B. dendrobatidis EGF83417.1 sequences group together with the animal C1GLT sequences. We provide all Fringe-like accessions in Supplementary Table S1 despite unresolved specificity questions.

**Additional file 2**. Phylogenetic trees for fungal fucosyltrasferases.

**Additional file 3**. Supplementary Table S1.

## Data Availability

All accessions and assemblies are listed in Additional file 3: Table S1.

## Abbreviations

GlcNAc: N-acetylglucosamine
EDF: Early-diverging fungi
FtoL: Fungal tree of life
DMT: Drug/metabolite transporter
ABC: ATP-binding cassette
AAL: Aleuria aurantia lectin
LFNG: O-fucosylpeptide 3-beta-N-acetylglucosaminyltransferase
TPM: Transcripts per million reads
EPS: Exopolysaccharides
