# Supplementary material for: Fucose as a nutrient ligand for Dikarya and a building block of early diverging lineages
Source: IMA Fungus. 2023 Sep 5;14:17. doi: 10.1186/s43008-023-00123-8 (PMC10481521; doi:10.1186/s43008-023-00123-8)
Supplement: Supplementary file 1 — Additional file 1. CLANS clustering of GT-A clan peptidyltransferases present in Fungi with animal representatives of transferases acting on glucosylated, fucosylated and galactosylated peptides. After clustering of fungal Fringe homologs with 42 animal representatives of both LFNG and C1GLT transferases, we obtained four clusters with 4946 fungal sequences. However, only four fungal sequences (Rozella allomycis RKP19558.1,RKP21212.1, EPZ36320.1 and Fusarium oxysporum EXK84998.1) group together with the human LFNG (Q8NES3) protein sequence. Two major fungal clusters are equally distant from LFNG and C1GLT clans. Basidiobolus meristosporus ORX91553.1 and Batrachochytrium salamandrivorans KAH6567989.1, KAH6579126.1 and B. dendrobatidis EGF83417.1 sequences group together with the animal C1GLT sequences. We provide all Fringe-like accessions in Supplementary Table S1 despite unresolved specificity questions. [file 43008_2023_123_MOESM1_ESM.pdf]

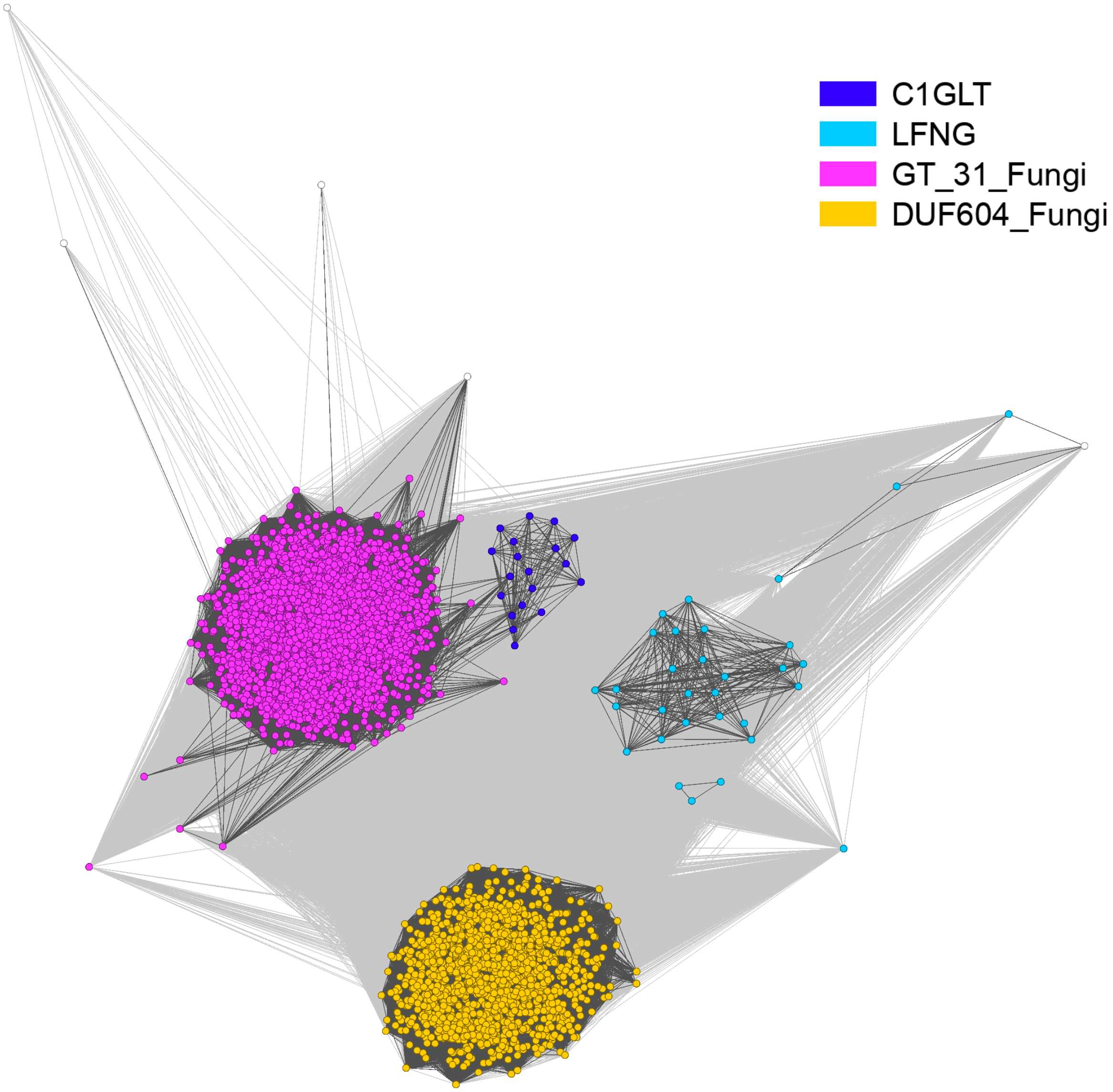

Supplementary Figure 1. CLANS clustering of GT-A clan peptidyltransferases present in Fungi with animal representatives of transferases acting on glucosylated, fucosylated and galactosylated peptides. After clustering of fungal Fringe homologs with 42 animal representatives of both LFNG and C1GLT transferases, we obtained four clusters with 4946 fungal sequences. However, only four fungal sequences (*Rozella allomycis* RKP19558.1,RKP21212.1, EPZ36320.1 and *Fusarium oxysporum* EXK84998.1) group together with the human LFNG (Q8NES3) protein sequence. Two major fungal clusters are equally distant from LFNG and C1GLT clans. *Basidiobolus meristosporus* ORX91553.1 and *Batrachochytrium salamandrivorans* KAH6567989.1, KAH6579126.1 and *B. dendrobatidis* EGF83417.1 sequences group together with the animal C1GLT sequences. We provide all Fringe-like accessions in Supplementary Table S1 despite unresolved specificity questions.
